# Investigating the effects of Pilates exercises on menopausal symptoms and sexual dysfunction in postmenopausal women: A randomized controlled trial

**DOI:** 10.1097/MD.0000000000042689

**Published:** 2025-06-06

**Authors:** Merve Yiğit Kocamer, Esra Atilgan

**Affiliations:** a Faculty of Health Sciences, Department of Physiotherapy and Rehabilitation, Istanbul Medipol University, Istanbul, Turkey.

**Keywords:** menopausal symptoms, Pilates, postmenopause, quality of life, sexual dysfunction

## Abstract

**Background:**

Menopause, a major transition in a woman’s life characterized by decline of estrogen and progesterone levels, is a hormonal shift associated with a range of symptoms that can adversely impact women’s quality of life, including vasomotor symptoms such as hot flushes and night sweats, and urogenital symptoms such as vaginal dryness and sexual dysfunction. This study aimed to investigate the effects of Pilates exercises on menopausal symptoms and sexual dysfunction in postmenopausal women.

**Methods:**

A total of 30 postmenopausal women, divided into a Pilates group and a control group, participated in this study. The Pilates group took part in an 8-week Pilates exercise program, while the control group received no exercise intervention, but attended menopause information sessions. The data were collected using the Menopause Rating Scale, the Menopausal-Specific Quality of Life scale, and the Female Sexual Function Index before and after the intervention. Body mass index was recorded, but not used in the primary analysis.

**Results:**

Post-intervention scores on the Menopause Rating Scale and Menopausal-Specific Quality of Life scales indicated better management of menopausal symptoms and an enhancement in the quality of life in the Pilates group. The Female Sexual Function Index scores in the Pilates group also improved, suggesting a positive impact of Pilates exercise program on sexual function. Taken together, the findings demonstrate the potential of Pilates exercises as an effective non-pharmacological intervention for alleviating menopausal symptoms, enhancing quality of life, and improving sexual function in postmenopausal women.

**Conclusion:**

This study suggests that regular Pilates exercises can be an effective non-pharmacological intervention to alleviate menopausal symptoms and improve sexual dysfunction in postmenopausal women. The findings support the integration of Pilates into postmenopausal care strategies to enhance overall wellbeing among postmenopausal women.

## 1. Introduction

Menopause, a significant transition in a woman’s life characterized by the cessation of menstruation and a decline in ovarian function, leads to reduced levels of estrogen and progesterone. This hormonal shift is associated with a range of symptoms that can adversely affect women’s quality of life, including vasomotor symptoms such as hot flashes and night sweats, psychological symptoms like mood swings and depression, as well as urogenital symptoms such as vaginal dryness and sexual dysfunction. These symptoms, collectively referred to as menopausal symptoms, can vary in severity and duration and frequently require intervention to alleviate their impact on daily life. Sexual dysfunction during the postmenopausal period is particularly concerning, as it can lead to a decline in intimacy and overall life satisfaction. Estrogen deficiency is closely linked to urogenital atrophy, which can result in vaginal dryness, dyspareunia (painful intercourse), and a decrease in libido. The psychological impact of menopause, including feelings of the loss of femininity and aging, further complicates sexual health.^[1]^ Sexual health in women is conventionally evaluated using the Female Sexual Function Index (FSFI), which provides a comprehensive evaluation across domains such as desire, arousal, lubrication, orgasm, satisfaction, and pain. Previous research has consistently demonstrated that postmenopausal women report lower scores on the FSFI, indicating a higher prevalence of sexual dysfunction.^[2]^

Considering the complex interplay of physical and psychological symptoms during menopause, in recent years, non-pharmacological interventions have gained particular attention as complementary approaches to traditional hormone replacement therapy (HRT). Physical exercise, particularly mind–body practices like Pilates, has emerged as a potential therapeutic strategy to mitigate menopausal symptoms and improve overall wellbeing. Emphasizing core stability, flexibility, and controlled breathing, Pilates offers a holistic approach to enhancing both physical and mental health. The positive effects of Pilates on menopausal symptoms has been consistently reported in the literature. For instance, in a study on the impact of a 12-week Pilates program on menopausal symptoms in 60 postmenopausal women, Kalavathi and Prathiba found significant reductions in the severity of hot flashes, sleep disturbances, and psychological symptoms among participants who engaged in Pilates as compared to a control group. This suggests that regular participation in Pilates can help manage vasomotor and psychosocial symptoms associated with menopause.^[3]^ In another study on the effects of Pilates on sexual function in 45 postmenopausal women randomly assigned to either a Pilates intervention group or a control group, Tenório et al found that, after 12 weeks, the Pilates group showed significant improvements in sexual function, particularly in the domains of arousal, lubrication, and orgasm, as measured by the FSFI. The authors concluded that Pilates could be an effective non-pharmacological intervention to improve sexual health in postmenopausal women, offering a safe and accessible option for those experiencing sexual dysfunction. Further supporting these findings, a systematic review by de Araújo et al evaluated the impact of various forms of exercise on menopausal symptoms. The review included randomized controlled trials that evaluated the effects of aerobic exercise, resistance training, yoga, and Pilates.^[4]^ The results indicated that Pilates was particularly effective in reducing menopausal symptoms, improving physical fitness, and enhancing psychological wellbeing. The review also highlighted the need for more high-quality studies to confirm these benefits and explore the long-term effects of Pilates on menopausal health. In another relevant study on the impact of Pilates on bone density and body composition in 50 postmenopausal women, Lee et al found that that participants experienced not only a reduction in menopausal symptoms, but also an improvement in bone mineral density and a decrease in body fat percentage.^[5]^ These findings suggest that Pilates could also play a role in mitigating the risk of osteoporosis, a common concern for postmenopausal women due to the decline in estrogen levels.^[6]^

In a more recent study, Griera et al investigated the effects of Pilates on mood and anxiety in postmenopausal women. The study involved a 10-week Pilates program, with participants reporting significant reductions in anxiety levels and improvements in mood stability. These psychological benefits are particularly important considering the emotional challenges many women face during the menopausal transition. Overall, available literature underscores the potential of Pilates as a multifaceted intervention for managing menopausal symptoms. While hormone replacement therapy remains a standard treatment option, the risks associated with HRT have led many women to seek alternative or complementary therapies.^[7]^ In this context, Pilates offers a low-risk, accessible, and effective option to improve both physical and mental health during the postmenopausal period. However, while the benefits of Pilates are well-supported, further research is needed to explore its effects on a wider range of menopausal symptoms and to establish standardized protocols for its implementation.

Accordingly, the present study aims to contribute to the literature by specifically examining the effects of an 8-week Pilates program on menopausal symptoms and sexual dysfunction in postmenopausal women. By providing a comprehensive evaluation of the physical, psychological, and sexual health outcomes associated with Pilates, we seek to inform healthcare providers and postmenopausal women about the potential benefits of incorporating Pilates into their wellness routines.^[8]^

## 2. Materials and methods

### 2.1. Study design and participants

This study was designed as a randomized controlled trial to investigate the effects of Pilates exercises on menopausal symptoms and sexual dysfunction in postmenopausal women. A total of 30 postmenopausal women were recruited from a local community center. The inclusion criteria for participants were as follows: (1) being in the postmenopausal stage (defined as the cessation of menstruation for at least 12 months); (2) experiencing menopausal symptoms; and (3) age between 45 and 65 years old. The women who currently used HRT, had a history of chronic diseases (e.g., cardiovascular disease or diabetes), or had physical conditions that would contraindicate participation in Pilates exercises were excluded. The 30 participants were randomly assigned to either the Pilates intervention group (n = 15) or the control group (n = 15). Randomization was performed using a computer-generated randomization sequence to ensure equal distribution across the 2 groups.

Ethical approval for the study was obtained from the Ethics Committee of Medipol University with protocol number (E-10840098-202.3.02-1757), dated from March 2023 to May 2024. The study was planned in accordance with the Helsinki Declaration and registered under ClinicalTrials.gov with the registration number (NCT06837714).

### 2.2. Intervention

The Pilates intervention group participated in an 8-week Pilates exercise program. The program consisted of sessions held twice a week, each lasting approximately 60 minutes. The Pilates exercises included in the program focused on core stability, flexibility, balance, and controlled breathing, and were tailored to accommodate the participants’ fitness levels. Each session was conducted by a certified Pilates instructor who ensured proper technique and safety during the exercises. The control group did not engage in any structured exercise program during the 8-week period. Instead, they attended a menopause information session where they received education on menopause-related issues, including symptom management, lifestyle modifications, and general health tips for postmenopausal women.

### 2.3. Outcome measures

The primary outcome measures for this study were menopausal symptoms and sexual dysfunction. These outcomes were evaluated using validated scales administered at the following 2 time points: baseline (before the start of the intervention) and post-intervention (after 8 weeks).

Menopause Rating Scale (MRS) was used to evaluate the severity of menopausal symptoms experienced by the participants. The MRS covers a range of symptoms, including vasomotor, psychological, and urogenital symptoms. The participants rated the severity of each symptom on a Likert scale, with higher scores indicating more severe symptoms.

Menopausal-Specific Quality of Life scale (MENQOL) was used to measure the impact of menopausal symptoms on the participants’ quality of life across several domains, including physical, psychological, and sexual wellbeing. The MENQOL provides a comprehensive assessment of how menopausal symptoms affect daily life, with higher scores indicating a greater negative impact.

FSFI was used to evaluate sexual function, focusing on domains such as sexual desire, arousal, lubrication, orgasm, satisfaction, and pain. The participants were asked to rate their experiences in each domain over the past 4 weeks. Higher FSFI scores indicated better sexual function.

### 2.4. Anthropometric measurements

While body mass index (BMI) was recorded for both groups at baseline, it was not used as a primary outcome measure. The BMI of participants in the Pilates group was 27.82 ± 3.90, while the BMI of participants in the control group was 29.78 ± 3.33. These measurements were included in the demographic analysis to provide an overview of the participants’ physical characteristics.

### 2.5. Data analysis

The data were analyzed using X. Descriptive statistics such as age, BMI, and menopausal status were used to summarize the participants’ baseline characteristics. Paired *t* tests were conducted to compare pre- and post-intervention scores within each group, while independent *t* tests were used to compare the changes in outcome measures between the Pilates and control groups. A significance level of *P* < .05 was considered statistically significant for all analyses.

This structured and systematic approach ensured that the effects of the Pilates intervention on menopausal symptoms and sexual dysfunction were accurately assessed and compared to the control group, providing valuable insights into the potential benefits of Pilates for postmenopausal women.

## 3. Results

The data collected from both the Pilates and control groups were analyzed to evaluate changes in MRS, MENQOL, and FSFI scores before and after an 8-week intervention period. The findings revealed significant differences between the 2 groups, with the Pilates group showing marked improvements across all measured outcomes.

Specifically, the results revealed that a higher proportion of women in the Pilates group showed improvement across all 3 measures as compared to the control group (see Table 1). This finding suggests that Pilates exercises may be more effective in reducing menopausal symptoms, improving quality of life, and enhancing sexual function in postmenopausal women.

**Table 1:** Improvement proportion in each group.

	Pilates_Improvement	Control_Improvement
MRS	1.0	1.0
MENQOL	1.0	1.0
FSFI	0.066	0.0

FSFI = Female Sexual Function Index, MENQOL = Menopausal-Specific Quality of Life scale, MRS = Menopause Rating Scale.

**Figure 1.**
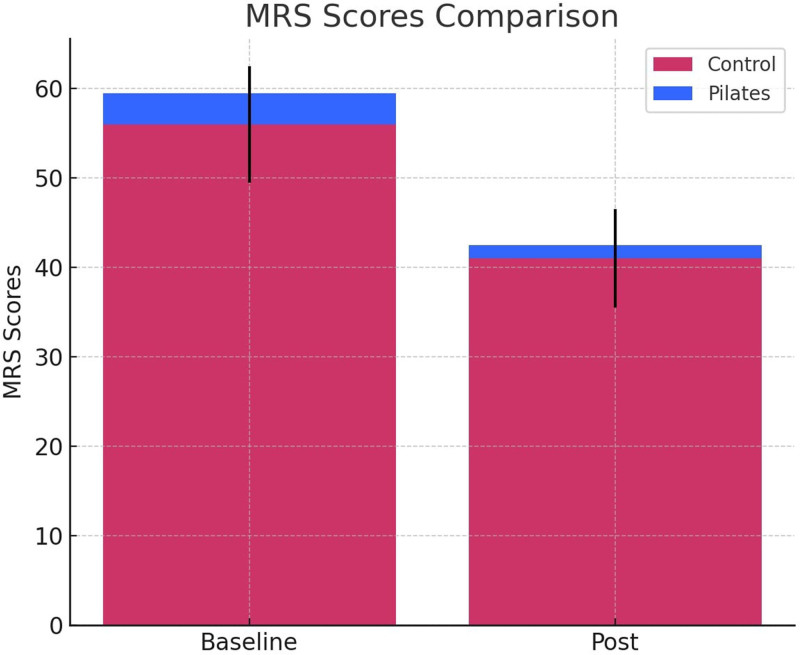
MRS scores comparison. MRS = Menopause Rating Scale.

The MRS scores of the participants in the 2 groups before and after the intervention are shown in Figure 1. The results revealed that the Pilates group showed a significant decrease in MRS scores post-intervention, indicating that Pilates exercises may be effective in alleviating menopausal symptoms. By contrast, the control group exhibited a less pronounced reduction in MRS scores, suggesting that menopausal symptoms were less alleviated in the absence of exercise.

Table 2 presents the standard deviation of changes in MRS, MENQOL, and FSFI scores for both the Pilates and control groups. A lower standard deviation in the control group across the measures suggests that changes in this group were more consistent. By contrast, the Pilates group exhibited greater variability, indicating that, while the intervention was generally beneficial, its effects varied more widely among participants.

**Table 2:** Standard deviation of changes in scores.

	Pilates_Std_Dev	Control_Std_Dev
MRS	11.197	6.927
MENQOL	8.590	9.093
FSFI	6.374	5.565

FSFI = Female Sexual Function Index, MENQOL = Menopausal-Specific Quality of Life scale, MRS = Menopause Rating Scale.

To gain an insight into the central tendency of the score changes in each group, we also computed the median changes in scores (see Table 3). The Pilates group demonstrated larger median improvements in MRS and MENQOL scores, suggesting a substantial reduction in menopausal symptoms and an improvement in quality of life for most participants. The median change in FSFI scores was also higher in the Pilates group, indicating an overall positive shift in sexual function.

**Table 3:** Median changes in scores.

	Pilates_Median_Change	Control_Median_Change
MRS	‐18.337	‐17.266
MENQOL	‐20.978	‐19.893
FSFI	11.512	8.260

FSFI = Female Sexual Function Index, MENQOL = Menopausal-Specific Quality of Life scale, MRS = Menopause Rating Scale.

**Figure 2.**
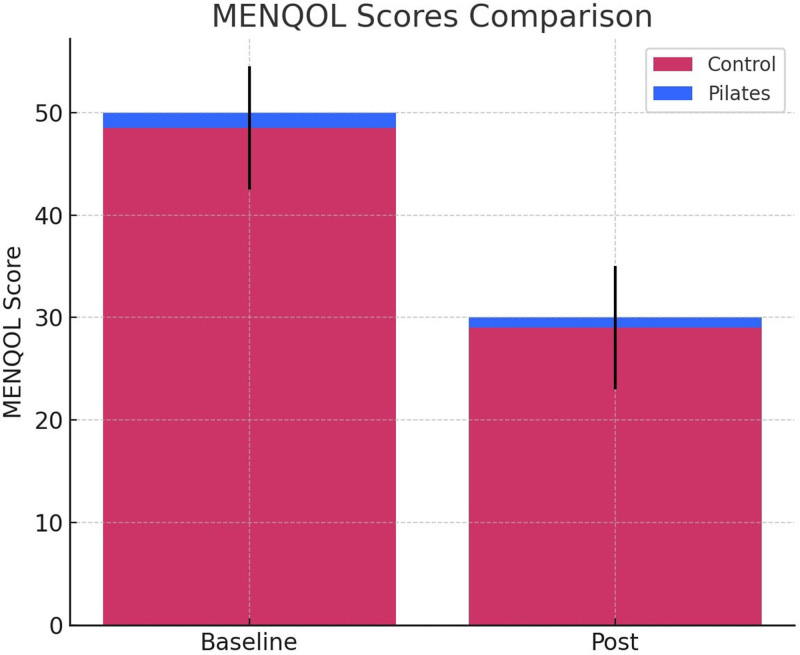
MENQOL scores comparison. MENQOL = Menopausal-Specific Quality of Life scale.

Figure 2 illustrates the MENQOL scores for the Pilates and control groups before and after the intervention. As can be seen in the figure, the Pilates group demonstrated a significant improvement in MENQOL scores post-intervention, highlighting the potential of Pilates to reduce the negative impact of menopausal symptoms on quality of life. The control group showed a more modest improvement, suggesting that Pilates exercises may positively contribute to enhancing quality of life during menopause.

**Figure 3.**
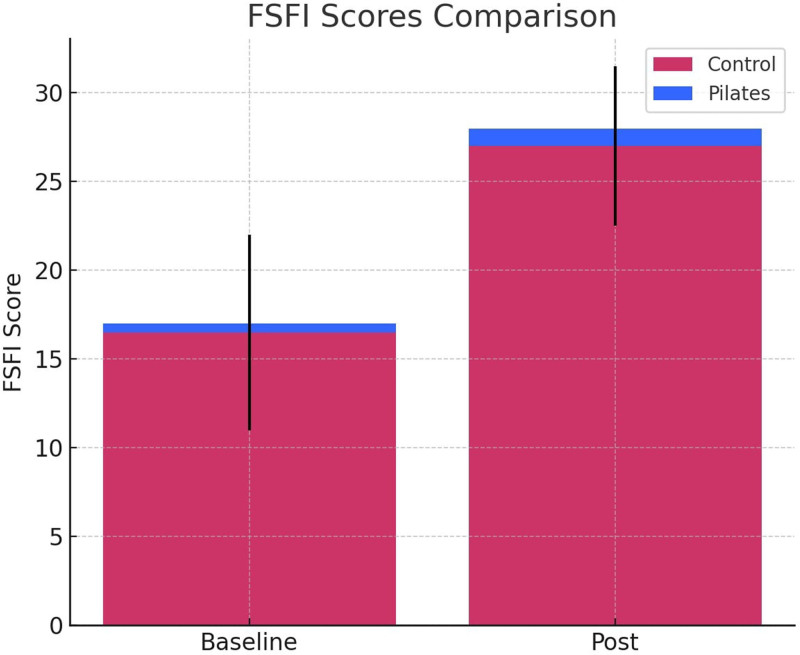
FSFI scores comparison. FSFI = Female Sexual Function Index.

Figure 3 compares the FSFI scores of the 2 groups before and after the intervention. The Pilates group showed a notable increase in FSFI scores post-intervention, indicating that Pilates exercises may improve sexual function in postmenopausal women. To compare, the control group showed a more limited increase in FSFI scores, suggesting that Pilates may play a significant role in enhancing sexual function during the postmenopausal period.

Table 4 highlights the range of changes in MRS, MENQOL, and FSFI scores, reflecting the spread between the maximum and minimum changes observed in each group. The Pilates group showed a broader range of changes, particularly in MRS and FSFI scores, suggesting that, while some of the participants experienced significant benefits, others witnessed more modest changes. The narrower range in the control group indicates less variation in outcomes, reinforcing the conclusion that the effects of the intervention were more pronounced in the Pilates group.

**Table 4:** Range of changes in scores.

	Pilates_Range	Control_Range
MRS	32.153	23.890
MENQOL	27.380	26.358
FSFI	24.155	21.026

FSFI = Female Sexual Function Index, MENQOL = Menopausal-Specific Quality of Life scale, MRS = Menopause Rating Scale.

**Figure 4.**
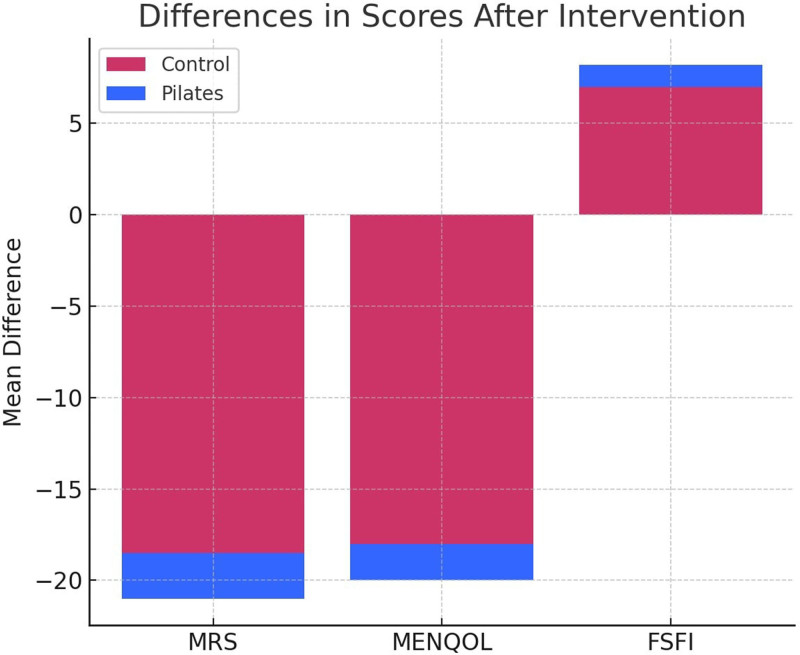
Differences in scores after intervention.

Figure 4 compares the differences between the 2 groups in MRS, MENQOL, and FSFI scores after the intervention. The Pilates group exhibited greater differences in all 3 scales (MRS, MENQOL, and FSFI), indicating that Pilates exercises are more effective in reducing menopausal symptoms, improving quality of life, and enhancing sexual function. By contrast, the control group showed smaller differences, emphasizing the notable impact of Pilates.

## 4. Discussion

In this study, we explored the effects of Pilates exercises on menopausal symptoms, quality of life, and sexual function in postmenopausal women. The results provide compelling evidence that Pilates can be a valuable non-pharmacological intervention to manage the multifaceted challenges associated with menopause. The data revealed significant improvements in the Pilates group across various measures, including the MRS, the MENQOL, and the FSFI. Our findings are consistent with previous studies demonstrating the important role of physical exercise, particularly mind–body practices like Pilates, in enhancing postmenopausal women’s wellbeing.^[9]^

The results of improvement proportion analysis highlighted that, as compared to the control group, a significantly higher proportion of participants in the Pilates group experienced improvements in their MRS, MENQOL, and FSFI scores. This finding suggests that regular engagement in Pilates exercises may effectively reduce the severity of menopausal symptoms, improve quality of life, and enhance sexual function. These improvements can be attributed to the holistic nature of Pilates, which not only strengthens the body, but also promotes mental and emotional wellbeing through controlled breathing, concentration, and relaxation techniques. The positive impact on MRS and MENQOL scores also underscores the potential of Pilates to address the physical and psychological dimensions of menopause, making it a promising alternative or complementary approach to traditional treatments like hormone replacement therapy.^[10]^

Furthermore, the results of the standard deviation analysis showed that, while the Pilates group showed greater overall improvement, there was also greater variability in the outcomes as compared to the control group. This variability suggests that, while Pilates can be highly effective for some women, its impact may vary depending on individual factors such as baseline fitness levels, adherence to the exercise regimen, and psychological readiness for lifestyle changes. These findings are consistent with previous mixed results concerning the effectiveness of exercise interventions for menopausal symptoms, highlighting the need for personalized approaches when recommending exercise as a treatment modality.^[11]^

In addition, the median changes in the FSFI scores further reinforced our conclusion about the positive effects of Pilates on menopausal symptoms and quality of life. While the Pilates group exhibited larger median improvements in both MRS and MENQOL scores, suggesting that most participants benefited from the intervention, the improvement in FSFI scores, although more modest, was also noteworthy, particularly if we consider the complexity of addressing sexual dysfunction in postmenopausal women. These results align with previous research showing the benefits of Pilates for improving flexibility, core strength, and mental focus, all of which can contribute to a better overall quality of life during menopause.

Additional insight into the differential impact of Pilates was obtained from the results on the range of changes in MRS and FSFI scores. Specifically, the broader range of changes observed in the Pilates group, particularly in MRS and FSFI scores, highlights that while some participants experienced significant benefits, others had more moderate or even minimal improvements. This finding highlights the importance of individualizing exercise programs to maximize benefits and address specific needs. It also raises important questions about the factors that may influence individual variability in response to Pilates, such as prior experience with exercise, motivation levels, and the presence of other health conditions.^[12]^

Taken together, the results of this study provide important implications for clinical practice and future research. First, they underscore the potential of Pilates as a low-risk, accessible, and effective intervention to manage menopausal symptoms. In the context of the growing concerns about the long-term safety of hormone replacement therapy, Pilates offers a viable alternative that can be easily incorporated into daily routines. Moreover, the mental and emotional benefits of Pilates, as reflected in the improved MENQOL scores, make it particularly valuable for women who experience mood disturbances, anxiety, and other psychological symptoms during menopause. Accordingly, future research should focus on exploring the mechanisms underlying the variability in response to Pilates, with a particular emphasis on identifying predictors of success. Longitudinal studies that would track the effects of Pilates over extended periods of time would be particularly valuable in determining the long-term benefits and sustainability of the improvements observed in this study. In addition, further investigation into the impact of Pilates on sexual function, as measured by the FSFI, is warranted, particularly in the light of the modest improvements observed in this area. Understanding how Pilates influences sexual health could lead to the development of targeted interventions addressing this frequently overlooked aspect of women’s health during menopause.^[13]^

Overall, this study adds to the growing body of evidence supporting the use of Pilates as an effective intervention for managing menopausal symptoms, improving quality of life, and enhancing sexual function in postmenopausal women. The positive outcomes observed in the Pilates group, despite the observed variability in response, highlight the potential of this exercise modality to provide significant health benefits during a challenging phase in women’s life. In the context of the growing awareness about the importance of holistic health approaches, Pilates is likely to become an increasingly important tool in the management of menopause and the promotion of overall wellbeing in postmenopausal women.^[14]^

It should also be noted that that there are other treatment strategies providing similar results to those reported in the present study. One of these treatment strategies with proven efficacy and safety is fractional CO_2_ laser therapy. This therapy was reported to improve quality of life and menopausal symptoms in the treatment of vulvo-vaginal atrophy in postmenopausal patients,^[15]^ as well as to positive impact clinical symptoms and sexual function in menopausal gynecological cancer survivors.^[16]^ Another study that involved a noninvasive treatment of vulvo-vaginal atrophy with CO_2_ laser in menopause found that microablative CO_2_ laser therapy caused morphological changes in vaginal tissues, thereby improving genitourinary menopause syndrome and positively affecting the quality of life and sexual life of postmenopausal women.^[17]^ Similarly, in a study reporting that urinary incontinence, which increases with age, negatively impacts women’s quality of life, an updated overview of clinical practices and surgical procedures for urinary incontinence was presented and new strategies to improve quality of life were evaluated.^[18]^

## 5. Conclusion

The results of the present study demonstrate the potential of Pilates exercises as an effective non-pharmacological intervention to alleviate menopausal symptoms, enhance quality of life, and improve sexual function in postmenopausal women. These findings indicate significant improvements in the Pilates group as compared to the control group, particularly in the reduction of menopausal symptoms and the enhancement of overall wellbeing. Despite some variability in individual responses, the overall positive outcomes suggest that Pilates can be a valuable component of a comprehensive approach to menopause management.

Pilates offers a holistic method that addresses both the physical and psychological challenges associated with menopause, making it a promising alternative or complement to traditional treatments. As a low-risk, accessible form of exercise, Pilates can be easily integrated into daily routines, providing lasting benefits for women’s health during the postmenopausal period. Future research should focus on studying long-term effects of Pilates, as well as seek to better understand the factors that influence individual responses and its impact on sexual function. Such research would contribute to the development of more personalized and effective strategies to support women’s health and wellbeing throughout menopause and beyond.

## Acknowledgments

The authors thank the subjects for their time and effort.

## Author contributions

**Conceptualization:** Esra Atilgan.

**Project administration:** Merve Yiğit Kocamer.

**Resources:** Merve Yiğit Kocamer.

**Software:** Merve Yiğit Kocamer.

**Validation:** Esra Atilgan.

**Visualization:** Esra Atilgan.

**Writing – original draft:** Merve Yiğit Kocamer.

**Writing – review & editing:** Merve Yiğit Kocamer.
